# Bacterial c-di-GMP has a key role in establishing host–microbe symbiosis

**DOI:** 10.1038/s41564-023-01468-x

**Published:** 2023-08-31

**Authors:** Nancy Obeng, Anna Czerwinski, Daniel Schütz, Jan Michels, Jan Leipert, Florence Bansept, María J. García García, Thekla Schultheiß, Melinda Kemlein, Janina Fuß, Andreas Tholey, Arne Traulsen, Holger Sondermann, Hinrich Schulenburg

**Affiliations:** 1 Department of Evolutionary Ecology and Genetics, University of Kiel, Kiel, Germany; 2 Department of Systematic Proteome Research and Bioanalytics, University of Kiel, Kiel, Germany; 3 Max Planck Institute for Evolutionary Biology, Plön, Germany; 4 CSSB Centre for Structural Systems Biology, Deutsches Elektronen-Synchrotron DESY, Hamburg, Germany; 5 Institute of Clinical Molecular Biology, University of Kiel, Kiel, Germany; 6 Section of Biology, University of Kiel, Kiel, Germany; 7 Present Address: Institute of Toxicology and Pharmacology, University of Kiel, Kiel, Germany

**Keywords:** Experimental evolution, Bacterial evolution, Symbiosis, Bacterial genomics

## Abstract

Most microbes evolve faster than their hosts and should therefore drive evolution of host–microbe interactions. However, relatively little is known about the characteristics that define the adaptive path of microbes to host association. Here we identified microbial traits that mediate adaptation to hosts by experimentally evolving the free-living bacterium *Pseudomonas lurida* with the nematode *Caenorhabditis elegans* as its host. After ten passages, we repeatedly observed the evolution of beneficial host-specialist bacteria, with improved persistence in the nematode being associated with increased biofilm formation. Whole-genome sequencing revealed mutations that uniformly upregulate the bacterial second messenger, cyclic diguanylate (c-di-GMP). We subsequently generated mutants with upregulated c-di-GMP in different *Pseudomonas* strains and species, which consistently increased host association. Comparison of pseudomonad genomes from various environments revealed that c-di-GMP underlies adaptation to a variety of hosts, from plants to humans. This study indicates that c-di-GMP is fundamental for establishing host association.

## Abstract (web summary)

The bacterial second messenger c-di-GMP is important for host association of microbes in *C. elegans* and beyond.

## Main

**Fig. 1:**
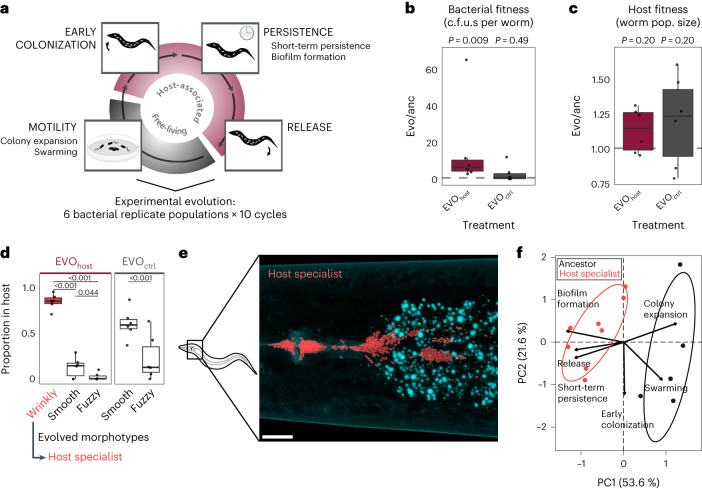
Microbiota bacteria evolve a host-specialist phenotype. **a**, Host-associated microbes transition from a free-living phase to host association, the latter comprising host entry, persistence and release. Six *P. lurida* populations were passaged ten times across these stages with the host *C. elegans* (EVO_host_) or without the host as a control (EVO_ctrl_). **b**, Host-adapted bacterial populations significantly increased fitness (given as c.f.u.s per worm) relative to the ancestor (two-sided *t*-tests and FDR-corrected Tukey post hoc comparisons, 6 replicates per treatment). **c**, Evolved bacteria remain beneficial to the host, determined by nematode population growth (two-sided *t*-test, 6 replicates per treatment). **d**, A wrinkly colony morphotype only emerged during host adaptation and dominates within worms (comparison of morphotype abundance within treatments: generalized linear models and FDR-corrected Tukey post hoc test, 6 replicates per treatment). **e**, Evolved host-specialist bacteria (tagged with red fluorescent dTomato) colonize the worm gut (autofluorescent vesicles in nematode intestinal cells in cyan). Scale bar, 10 µm. **f**, PCA of key traits of the host-associated life cycle (in **a**) reveals a distinct profile for evolved wrinkly host specialists compared with ancestral bacteria (see Supplementary Table 5 for individual traits measurements). **b–d**, Boxplots show median (centre line), upper and lower quartiles (box limits) and the interquartile range (whiskers).

Host-associated microorganisms have important effects on the physiological functioning and fitness of their plant and animal hosts^1–3^. These host–microbiota interactions are often studied using a host-centric view, with a focus on microbiota-mediated host functions. This view neglects the important fact that most microbes evolve faster than their hosts due to their shorter generation times and higher mutation rates, and thus that fitness improvements for the microbes may disproportionately drive the associations^4^. An important step in the evolution of a host–microbe association is the emergence of a more specialized interaction that allows free-living bacteria to reliably enter the host, persist and finally be released into the environment to colonize new hosts (Fig. 1a)^4^. Thus far, little is known about the traits and molecular processes that determine how bacteria adapt to such an association with the host.

## Results

### Evolution of host-specialist bacteria

**Extended Data Fig. 1:**
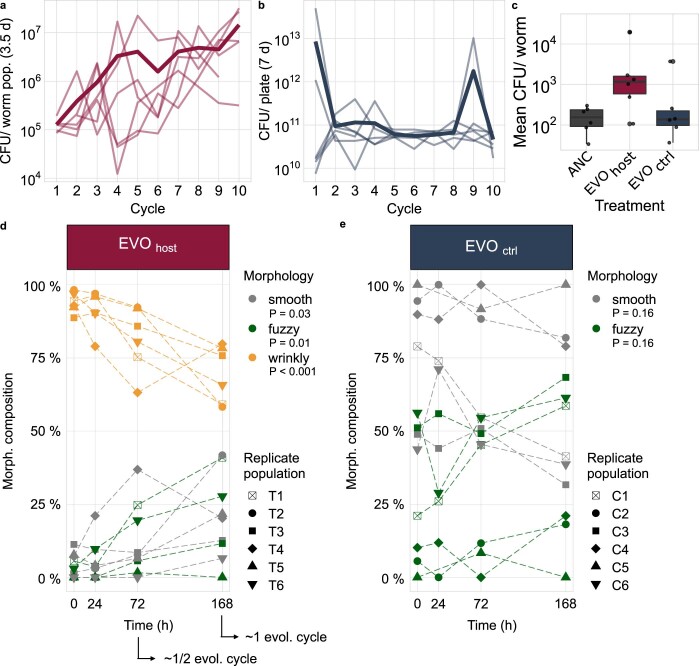
Bacterial fitness during and resulting from experimental evolution. **a, b, Bacterial fitness during the evolution experiment**. a, Bacterial fitness in host across cycles of the evolution experiment measured as colony forming units (CFU) per worm population after 3.5 days of exposure to *Ce_*MY316. b, In the negative control, bacterial fitness was assessed on nematode growth agar in absence of the host. For each data point, bacteria were collected at the bottleneck time point of the noted cycle. Replicate populations (n = 6) are shown as separate thin lines, with the mean shown as a thick line. **c**, Mean CFU per individual host in a worm population for the evolved bacterial populations of cycle 10. Five L4 *C. elegans* larvae proliferated on evolved or ancestral bacterial lawns for 3.5 days (reaching F1 generation) and CFUs were extracted from the whole worm population. CFUs per population were divided by the number of worms in the population. Overall, results are shown as boxplots, with boxes indicating 25% above and below the median, which is given as the thick line within boxes; replicate populations (n=6) are indicated as individual data points. **d, e**, Dynamic changes in morphotype composition during the free-living phase of the host-associated life cycle for bacterial populations from the end of the evolution experiment. c, Results for the replicate populations from the host-associated evolution treatment. Box plots show median (center line), upper and lower quartiles (box limits) and the interquartile range (whiskers). d, Results for the replicate populations from the control treatment. Proportions of the different colony morphotypes (see graphical legend) is shown across time of the host-associated life cycle. Time point 0 is at the end of the host-associated phase, when bacteria are transferred to the free-living phase, which itself lasts 168 hours. Fdr-corrected beta-regressions were used to predict proportions and test for a change in proportions over time (see Supplementary Table 2).

We studied the evolutionary transition from free-living to host association through controlled experimental evolution, using the bacterium *Pseudomonas lurida* and the nematode host *Caenorhabditis elegans* as a model. This bacterium is occasionally found in the natural microbiota of *C. elegans*^5,6^. Under laboratory conditions, the presence of *P. lurida* is associated with increased population growth rates of *C. elegans* and can provide protection against pathogens, yet both host and bacterium can proliferate without each other and thus do not depend upon one another^5,7,8^. To select host-adapted bacteria, we serially passaged 6 *P. lurida* populations either with or without a host-associated phase (Fig. 1a; EVO_host_ or EVO_ctrl_, respectively). All populations were inoculated from the same clonal ancestor. After 10 passages through hosts, the bacteria reached on average 5–10 times higher bacterial load in the host than their ancestor, a significant change not observed for the control that evolved without exposure to hosts but otherwise had identical conditions (Fig. 1b and Extended Data Fig. 1). The increased bacterial fitness did not come at a cost to the host, as nematode population growth (used as a proxy for nematode fitness^5^) did not change significantly, but rather increased in the presence of the adapted bacteria (Fig. 1c).

**Extended Data Fig. 2:**
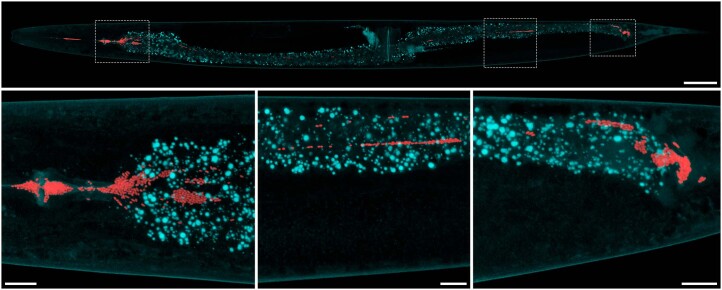
Colonization of the *C. elegans* intestine by wrinkly host specialists. Confocal laser scanning micrographs (upper panel: longitudinal optical section; lower panels: maximum intensity projections showing longitudinal optical sections) revealing intact bacterial cells (red) within the intestinal system of a young adult *Ce_*MY316 (cyan). The upper micrograph shows an overview of the complete worm, and the lower micrographs show detailed views of the worm sections indicated by the dashed frames above. These include the posterior pharynx with the worm grinder and the first intestinal ring (left), a central intestinal (middle) and the anal region (right). The bottom left panel is identical to the micrograph shown in the main text (Fig. 1e). Scale bars = 50 µm (overview) and 10 µm (detailed views).

As a result of passaging, bacterial populations diversified in colony morphology. At the end of our experiment a ‘wrinkly’ morphotype was dominant in all host-associated experimental replicate populations and absent in the controls, whereas ‘fuzzy’ and ‘smooth’ (ancestral) morphotypes were present across treatments (Fig. 1d and Supplementary Table 1). Despite their significant advantage in hosts, the wrinkly morphotypes declined during growth on agar, while smooth and fuzzy types increased in abundance (Extended Data Fig. 1 and Supplementary Table 2). As the wrinkly types were unique to and reached very high abundance in worm-adapted bacteria, we considered them host specialists. These specialists can be found in clusters within the intestinal tract of the nematode, especially in the anterior and posterior parts (Fig. 1e and Extended Data Fig. 2). Notably, the evolved wrinkly morphotype is similar to wrinkly *P. fluorescens* that emerge at the air–liquid interface in static microcosms^9^ and to rugose variants of various pathogenic bacteria^10–12^. Our experiments suggest that this morphological change also occurs in beneficial bacteria adapting to host association. For a further characterization of these adaptations, we focused on 47 clones of the distinct and genetically stable morphotypes (Supplementary Table 3) isolated from the final populations of our evolution experiment.

### Host specialists have a distinct lifestyle

**Extended Data Fig. 3:**
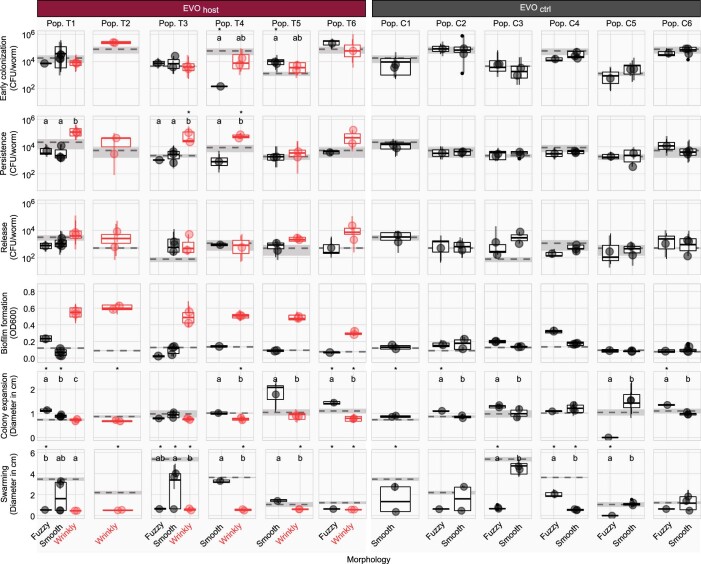
Wrinkly isolates from the end of the host-associated evolution treatment evolve a host-associated lifestyle. Phenotypes of morphotype clones isolated from independent host-evolved populations (left) and control populations (right), including smooth, fuzzy and wrinkly morphotypes, are shown. Results for each morphology are summarized as boxplots (1 < n < 4). Box plots show median (center line), upper and lower quartiles (box limits) and the interquartile range (whiskers). Dashed lines and grey shaded areas indicate the mean and standard error of ancestral traits, respectively. Differences between evolved morphologies and the ancestor were assessed with generalized linear models and fdr-corrected Tukey post-hoc tests. Letters indicate statistical differences between morphologies, asterisks indicate deviation from the ancestor (see Supplementary Table 6).

**Extended Data Fig. 4:**
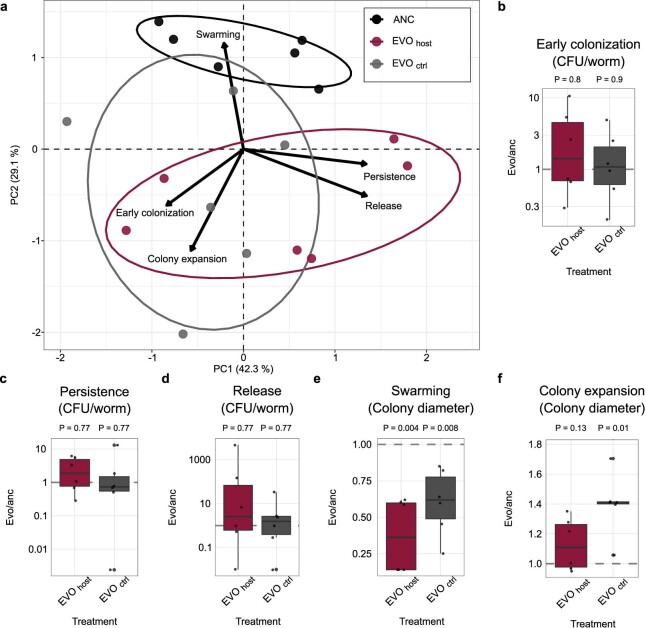
Evolution of a host-interaction life-style in the populations from the end of the host-associated evolution treatments. **a**, Principal component analysis on characteristic stages of host-association for ancestral, host evolved and control evolved bacterial populations. Individual data points refer to replicate populations colored according to evolution treatment (Supplementary Table 5). **b–f**, Shifts in phenotypes from the bacterial ancestor in the evolved populations for (**b**) early colonization, determined by CFU extracted from L4 larvae exposed to bacteria for 1.5 hour; (**c**) persistence in L4 larvae kept in M9 buffer for 1h (raised on bacteria); (**d**) CFU of bacteria released from L4 larvae into buffer within 1h (previously raised on bacteria from L1 to L4), (**e**) swarming distance on 0.5% agar within 24; and (**f**) colony expansion on 3.4% agar within 72h. All panels show ratios of evolved over ancestral populations for five replicates shown as individual data points. The dashed line indicates the mean values obtained for the ancestral population. The difference between evolved and ancestral phenotypes were assessed using one-sided t-tests (fdr-corrected; Supplementary Table 8). In all box plots, median (center line), upper and lower quartiles (box limits) and the interquartile range (whiskers) are shown.

An analysis of trait changes across the distinct stages of host association revealed specific adaptations of wrinkly morphotypes to the interaction with *C. elegans*. In detail, we characterized two traits of importance for the free-living stage and four traits for host association (as listed in Fig. 1a). We found that the wrinkly isolate profiles were significantly distinct from the ancestral trait profile (Fig. 1f and Supplementary Table 4). This was mainly due to significant increases in short-term persistence, release from the host and in vitro biofilm formation (Fig. 1f, Extended Data Fig. 3 and Supplementary Tables 4–6)—all traits that define late-phase interactions with the host. The overall pattern of improved host association was also recovered by analysing the genetically diverse populations from the end of the evolution experiment, where the host-associated populations similarly increased in persistence and release (Extended Data Fig. 4 and Supplementary Tables 7 and 8). In detail, biofilm formation can enable persistent contact with the host and increase stress tolerance^13,14^, as exemplified by many pathogens^15^, thereby improving survival in the nematode’s digestive tract. As a consequence of increased biofilm formation, aggregated cells may be expelled more easily^16^, thereby explaining the observed increase in release. Such shedding also enhances the chance for transmission to other hosts^4^, which restarts the cycle of host association. Notably, wrinkly isolates did not differ from ancestors in early colonization, yet showed a significant decrease in colony expansion and swarming on plates (Fig. 1f, Extended Data Fig. 3 and Supplementary Tables 4 and 5). The latter result is consistent with a decrease in motility described for *E. coli* that evolved to become a mutualist in stinkbugs^17^, but contrasts with findings that sufficient swarming is required for colonization initiation of zebrafish and bobtail squid^18,19^. These contrasts are probably due to differences in symbiont recruitment between the host systems, defined by either aquatic environments for zebrafish and squid, or terrestrial environments for *C. elegans* and stinkbug. Moreover, our observations of increased biofilm formation and reduced motility may indicate an evolved life-history trade-off between the traits defining host association and the free-living stage. We conclude that experimental evolution in the presence of the nematode host leads to the emergence and spread of a host-specialist type. We next asked whether the improved host association has a common genetic basis.

### c-di-GMP determines host specialization

**Fig. 2:**
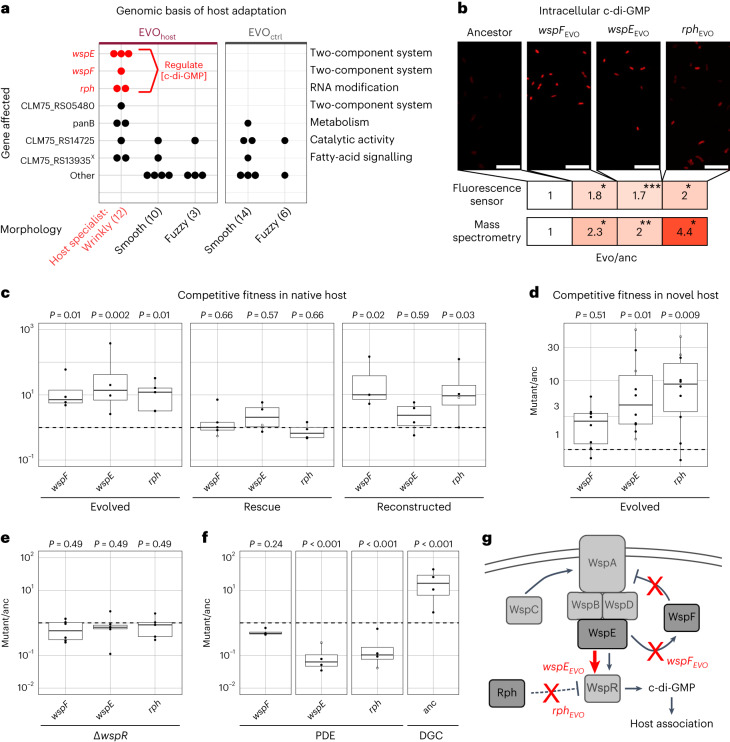
Wrinkly host specialists adapt to *C. elegans* by upregulation of the bacterial second messenger c-di-GMP and increased intra-host competitiveness. **a**, Overview of genes with non-silent changes in evolved bacterial isolates. Data points represent mutant isolates with one or multiple mutations in a given gene (the total number of isolates with a given morphology is specified in brackets). A cross indicates genes with variants lost in the evolved isolates as compared with the ancestor. **b**, Fluorescence sensor and LC–MS detected higher intracellular c-di-GMP concentrations in evolved wrinkly mutants compared with the ancestor (Welch’s ANOVA and Games–Howell post hoc comparisons, 5 replicates per treatment). Scale bars, 10 µm. **c**, Competitive fitness (c.f.u.s per worm relative to ancestor, dashed line) of evolved *wspF*, *wspE* and *rph* mutants (left), rescued mutants (middle) and reconstructed mutants in ancestral background (right) during persistence in *C. elegans* MY316 (3 < *n* < 5). **d**, Competitive fitness of evolved *wspF*, *wspE* and *rph* mutants during persistence in the non-native *C. elegans* strain N2 (8 < *n* < 10). **e**, Competitive fitness of *wspF*, *wspE* and *rph* mutants (5 < *n* < 6) with additional Δ*wspR* mutation compared to ancestral *Pl*_MYb11 (dashed line). **f**, Competitive fitness of *wspF*, *wspE* and *rph* expressing heterologous phosphodiesterase (PDE) PA2133 from plasmid (pJN2133) and ancestral MYb11 expressing constitutively active diguanylate cyclase (DGC) GCN4-WspR from plasmid (pJStrep-GCN4-WspR), each compared to their respective empty vector control (*n* = 4, dashed line). **c**–**f**, Persistence competition experiments were performed with ≥3 replicates per treatment and analysed with ANOVA (**d**) or LMM and FDR-corrected Dunnett post hoc tests; *P < 0.05, **P < 0.01, ***P < 0.001. Boxplots show median (centre line), upper and lower quartiles (box limits) and the interquartile range (whiskers). **g**, Graphical hypothesis of adaptive c-di-GMP manipulation via Rph and the Wsp system. Solid lines indicate previously established regulatory interactions, dashed lines emerging hypotheses. Red indicates the inferred consequences of the studied mutated gene from experimental evolution.

**Extended Data Fig. 5:**
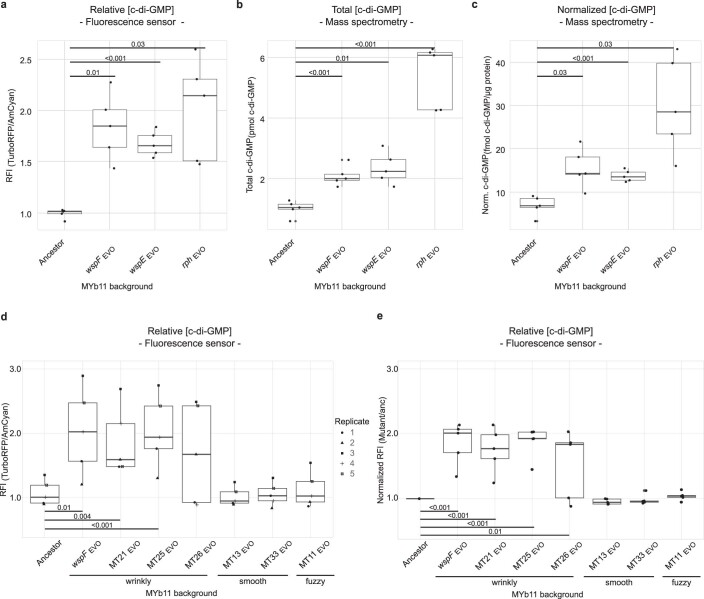
Increased intracellular c-di-GMP concentrations in wrinkly isolates. **a**, Amount of intracellular c-di-GMP measured with a fluorescence sensor. Raw fluorescence intensity (RFI) is the ratio of TurboRFP (c-di-GMP-dependent) and AmCyan (plasmid copy number-dependent) and, thus normalized for copy number of the sensor plasmid. **b**, Total c-di-GMP determined by isotope dilution PRM analysis. **c**, C-di-GMP determined by isotope dilution PRM analysis and normalized by total protein amount. In **a, b** and **c**, c-di-GMPs levels are studied for the ancestor and three wrinkly isolates from the end of the evolution experiment, each with a single mutation in either *wspF*, *wspE*, or *rph*. We compared c-di-GMP levels replicate cell populations (n = 5). **d**, Intracellular c-di-GMP of isolates from end the of the evolution experiment with wrinkly, smooth, and fuzzy colony morphology measured with a fluorescence sensor (nested ANOVA and fdr-corrected Dunnett post hoc test; n = 5). **e**, RFI of the different isolates normalized by ancestral RFI to correct for replication-dependent effects (ANOVA and fdr-corrected Dunnett post hoc test; n = 5; Supplementary Table 10. For respective mutations see Supplementary Table 9). Box plots show median (center line), upper and lower quartiles (box limits) and the interquartile range (whiskers).

**Extended Data Fig. 6:**
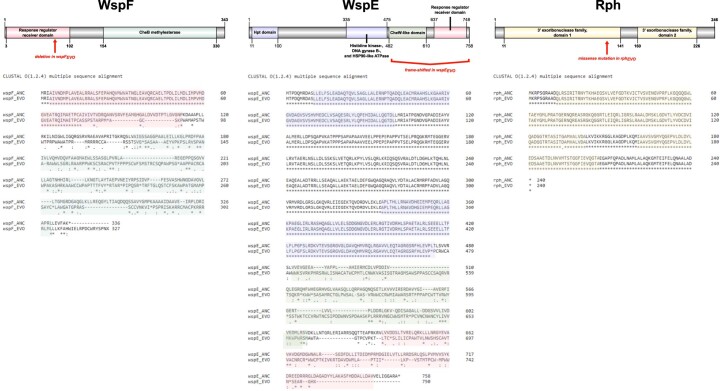
Amino acid sequence alignments of ancestral and evolved c-di-GMP regulating enzymes. On top, illustrations of protein domains show the organization of WspF, WspE and ribonuclease PH with sites affected by *Pl*_MYb11 mutation highlighted by red arrows. Below, protein sequence alignments of ancestral and evolved proteins are shown with domains highlighted in the same colors as above.

Whole-genome sequencing of the isolated morphotypes and the ancestor revealed several independent mutations in wrinkly host specialists that affect the bacterial second messenger cyclic diguanylate (c-di-GMP). In particular, a comparison of non-silent genomic variation identified variant genes specific to wrinkly host specialists (Fig. 2a and Supplementary Table 9). Two of the genes, *wspE* and *wspF*, code for a hybrid sensor histidine kinase and a methylesterase in the wrinkly spreader (*wsp*) operon, respectively^20^. These genes are part of a two-component system that regulates c-di-GMP levels (Fig. 2g) and wrinkly formation in beta- and gamma-proteobacteria, including pseudomonads^20–23^. We found additional mutations unique to the host specialists in the gene *rph*, encoding RNase PH that has not been linked to c-di-GMP signalling previously. Using both a fluorescence-based c-di-GMP sensor and liquid chromatography–mass spectrometry (LC–MS), we found a roughly twofold c-di-GMP increase in three wrinkly isolates, each with a single mutation in either *wspE*, *wspF* or *rph*, when compared with the ancestor (Fig. 2b, Extended Data Fig. 5 and Supplementary Table 10). This points to a loss-of-function mutation in *wspF* (which downregulates c-di-GMP) and alterations in active sites of WspE and Rph that all converge at upregulating c-di-GMP. We aligned evolved and ancestral amino acid sequences (Extended Data Fig. 6) and confirmed a disruption in WspF functional domains, as well as a disrupted receiver domain in WspE that probably prevents its de-autophosphorylation and thus constantly activates downstream WspR^24^. Amino acid substitutions in the exoribonuclease domain of Rph further link its ribonuclease activity to c-di-GMP metabolism. As we observed similar increases in c-di-GMP levels in other wrinkly, but not in smooth or fuzzy mutants (Extended Data Fig. 5), we subsequently asked whether the wrinkly-specific mutations indeed cause improved host association.

**Extended Data Fig. 7:**
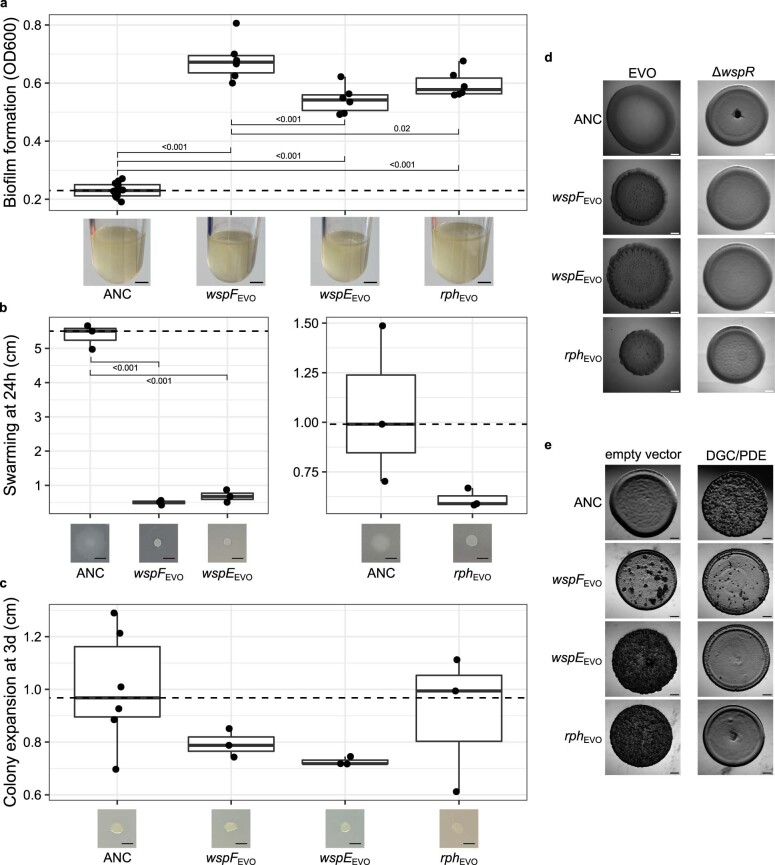
Biofilm formation and motility of focal wrinkly host specialists and macrocolonies of *Pl*_MYb11, evolved isolates, Δ*wspR* mutants and PDE/DGC expressing derivatives. **a**, Biofilm formation of *wspF*, *wspE*, and *rph* mutants compared to *Pl*_MYb11 (ancestral median = dashed line) after two days shaking incubation microtiter plates. Illustration of biofilms with photographs of biofilms from test tubes for after 48h incubation. Scale bars = 4mm. **b**, Swarming motility and **c**, colony expansion of isolates *wspF*, *wspE*, and *rph* mutants were compared to *Pl*_MYb11 (ancestral median = dashed line) after 24 and 72h, respectively. b-c, Scale bars = 0.5 cm. a-c, Experiments were performed with min. 3 replicates/treatment and analyzed with ANOVA and fdr-corrected Dunnett post-hoc tests or a one-sided t-test. Box plots show median (center line), upper and lower quartiles (box limits) and the interquartile range (whiskers). **d**, Macrocolonies of ancestral *Pl*_MYb11 and three wrinkly isolates from the end of the evolution experiment (with single mutations in either *wspF*, *wspE*, or *rph*). **e**, Macrocolonies of Δ*wspR* mutants in *Pl*_MYb11 *wspF*, *wspE*, or *rph* background. **f**, Macrocolonies of *wspF*, *wspE*, or *rph* isolates expressing the phosphodiesterase PA2133 from plasmid, *Pl_* MYb11 expressing the constitutively active diguanylate cyclase GCN4-WspR from plasmid, and empty vector controls. d-f, Macrocolonies were grown on tryptic soy agar supplemented with Congo Red for one (d,e) to three (f) days. Scale bars = 1mm.

**Extended Data Fig. 8:**
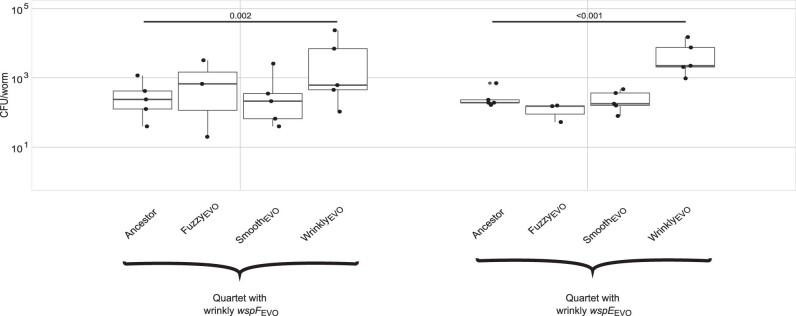
Increased competitive fitness of wrinkly isolates in bacterial mixtures of four strains (quartets). Three co-evolved morphotypes isolated from host-evolved replicate population T3 were paired with the ancestor. In one quartet the wrinkly *wspF* mutant (MT12) was present and in the other the wrinkly *wspE* mutant (MT14). Data points represent independent replicates, min. 3 replicates per treatment. Differences between morphotypes and ancestor were assessed using a linear mixed model and subsequent fdr-corrected Dunnett post-hoc comparisons; see Supplementary Table 13. Box plots show median (center line), upper and lower quartiles (box limits) and the interquartile range (whiskers).

A functional genetic analysis of *wspE*, *wspF* and *rph* demonstrated their direct involvement in host adaptation. For this analysis, we assessed the competitive fitness of mutants relative to the ancestor during host colonization. First, we re-assessed the three selected wrinkly mutants and found them to be significantly more competitive than the ancestor (Fig. 2c, left panel, and Supplementary Table 11), alongside increased biofilm formation and decreased swarming in vitro (Extended Data Fig. 7 and Supplementary Table 12). Thereafter, we rescued these mutants with the corresponding ancestral alleles, which indeed abolished the mutants’ fitness increase (Fig. 2c, middle panel, and Supplementary Table 11). Thirdly, an experimental introduction of each mutation into the ancestral background resulted in a significantly higher competitiveness, at least for the *wspF* and *rph* mutations (Fig. 2c, right panel, and Supplementary Table 11). A similar fitness advantage was observed for the *wspE* and *wspF* mutants when either was subjected to quartet competition with the ancestor and the two other morphotypes (Extended Data Fig. 8 and Supplementary Table 13). Notably, fitness advantages of evolved mutants were consistently observed in a non-native host strain (the *C. elegans* laboratory strain N2) (Fig. 2d and Supplementary Data Table 14). While two of these genes are components of the Wsp system, which regulates c-di-GMP during surface sensing in other pseudomonads^25,26^, *Pl*_MYb11 in theory possesses a variety of c-di-GMP modifying enzymes. This includes 34 genes coding for GGDEF and 22 coding for EAL domains with putative diguanylate cyclase (DGC) and c-di-GMP-specific phosphodiesterase (PDE) functions, respectively. We validated the role of the Wsp system’s cognate DGC in host adaptation using *wspR* knockouts in our evolved host-specialist mutants. This change abolished the mutants’ competitive advantage in the host (Fig. 2e) and caused a change from wrinkly to smooth colony morphology (Extended Data Fig. 7 and Supplementary Table 15), thus linking the DGC *wspR* to *wspE* and *wspF* (as expected) and *rph* (previously unknown). In addition, we directly manipulated c-di-GMP levels by heterologous expression of a PDE and a DGC from *P. aeruginosa*^23,27^, which respectively resulted in either decreased or improved persistence in *C. elegans*, as expected (Fig. 2f, Extended Data Fig. 7 and Supplementary Table 16). We thus conclude that changes in *wspE*, *wspF* and *rph* that converge on increasing c-di-GMP levels via the Wsp system enhance bacterial fitness in the host (Fig. 2g). As upregulation of this second messenger mediates a fundamental life-history switch^13^, we next investigated whether it more generally mediates host association across pseudomonads.

### c-di-GMP generally promotes symbiosis

**Fig. 3:**
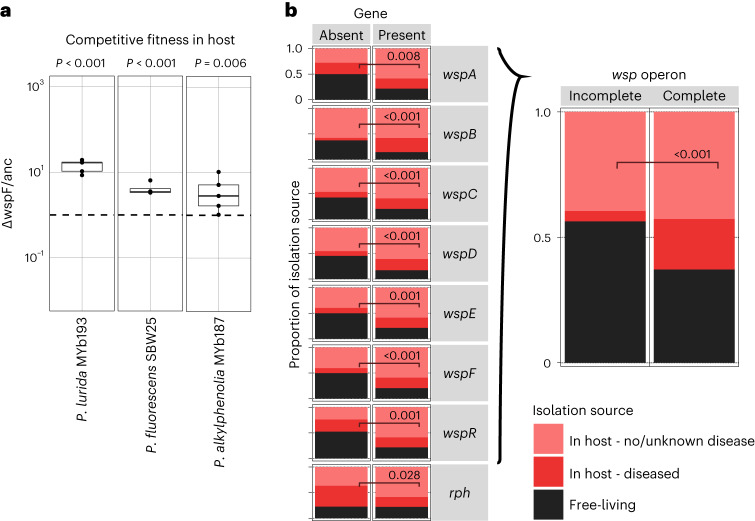
C-di-GMP regulators generally mediate host association across pseudomonads. **a,** *WspF* deletion increases intra-host competitive fitness of different *Pseudomonas* species relative to their wild type (c.f.u.s per worm, wildtype indicated by dashed line; LMM and FDR-corrected Dunnett post hoc tests, 3 < *n* < 5,). Boxplots show median (centre line), upper and lower quartiles (box limits) and the interquartile range (whiskers). **b**, Presence/absence of *wsp* genes and *rph*, as well as completeness of the *wsp* operon (that is, presence of *wspA-F* and *wspR*) co-vary with isolation source of sequenced pseudomonads (*Χ*^2^ goodness-of-fit tests).

**Extended Data Fig. 9:**
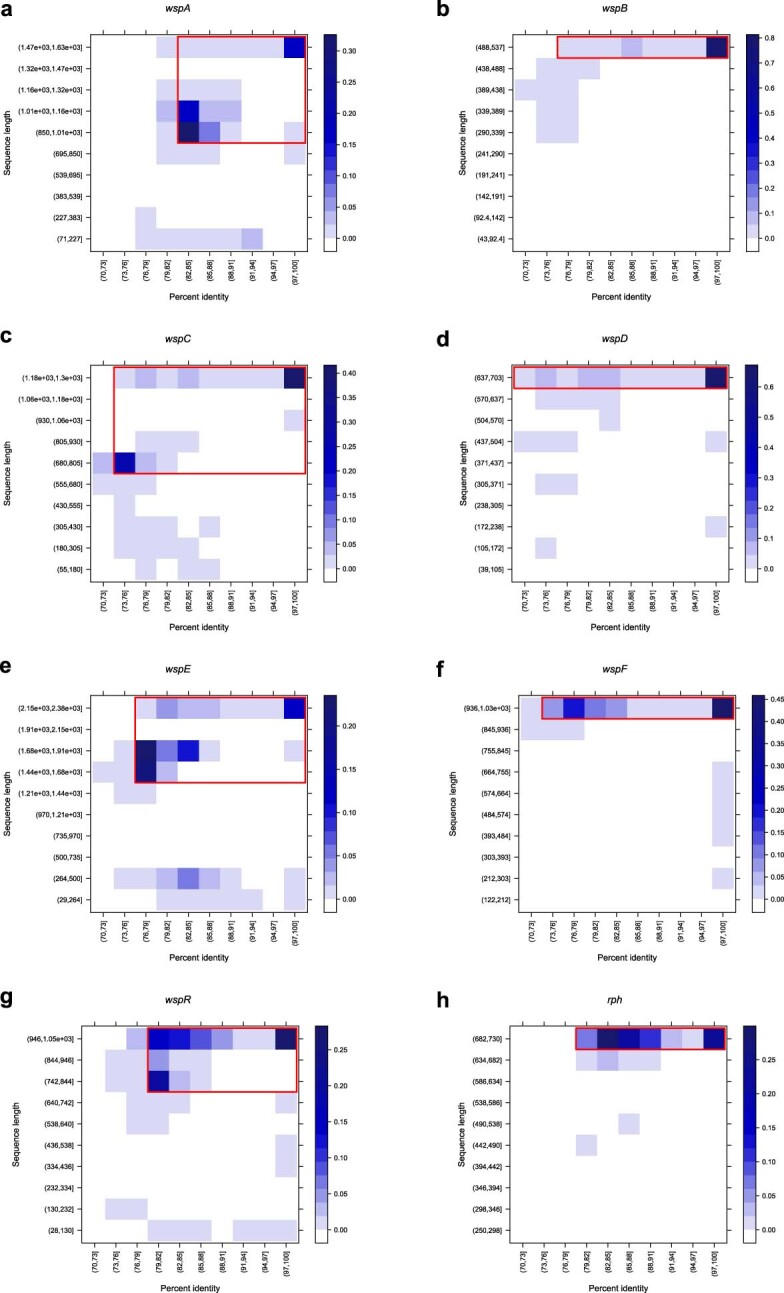
Filters for the identification of *rph* and *wsp* gene candidates. Distribution of sequence lengths and percent identities of the BLAST results for individual genes. The proportion of BLAST results belonging to particular sequence length and percent identity classes are shown as blue shades with varying intensity (cf. scale). Red rectangles show the areas for which the presence of the considered gene is assumed, and were set to include the largest BLAST hit values at both maximum sequence length and percent identities.

Genetic manipulation of *wspF* and a bioinformatic analysis of *Pseudomonas* genomes revealed a general involvement of *wsp* genes in host association. For the former, we generated *wspF* deletion mutants for *P. lurida* strain MYb193 and the distantly related *P. alkylphenolia* MYb187 (both naturally associated with *C. elegans*), and further obtained mutant and wildtype *P. fluorescens* strain SBW25, a model for wrinkly formation^21^. We found that the mutants had significantly higher competitive fitness in the *C. elegans* host than their respective wildtypes (Fig. 3a and Supplementary Table 17). Furthermore, we correlated the presence of *wsp* and *rph* genes in 1,359 whole *Pseudomonas* genomes from NCBI with the bacterial isolation source, a proxy for lifestyle (Extended Data Fig. 9 and Supplementary Table 18). *Pseudomonas* isolates containing any of the *wsp* genes or the complete, highly syntenic (Supplementary Table 19)^20^ *wsp* operon were significantly more often isolated from a host than isolates lacking these genes (Fig. 3b and Supplementary Table 19). These findings may seem surprising for genes with opposite regulatory effects (for example, *wspE* versus *wspF*), yet are probably explained by the syntenic inheritance of the entire operon with its set of interacting genes (Supplementary Table 19; see also ref. ^20^). Further, *rph* was more prevalent in isolates from healthy/undiagnosed hosts than from diseased hosts. Across lifestyles, we additionally detected signatures of negative selection for *wspE, wspF* and *rph*, which additionally suggest that they are functionally stabilized by selection when present (Supplementary Table 20). We propose that the presence of these genes allows the finetuned regulation of c-di-GMP and thereby, adjustment to a host-associated lifestyle.

## Discussion

**Extended Data Fig. 10:**
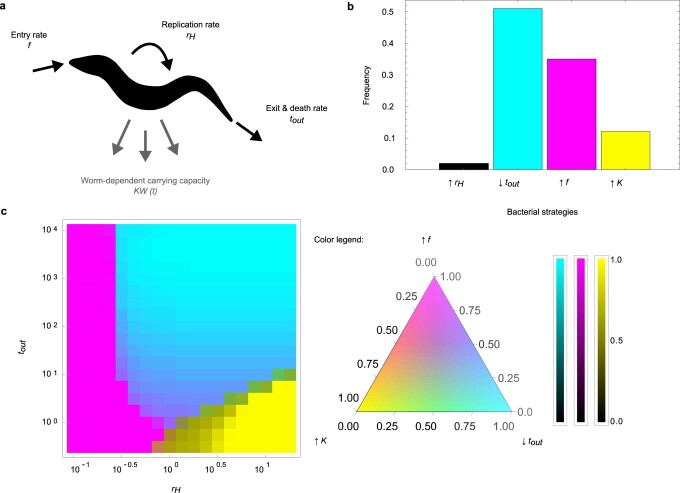
Model assessing the selection gradient on bacteria following a host-associated life cycle. **a**, Definition of the rates for the model of a microbial lineage being taken up by, replicating within and being expulsed from worms on a plate. **b**, Distribution of the optimal strategies across the whole traits space. **c**, Projection of the trait space over the axes (*r, δ*). For each point of the map, the color represents the proportions of times each of the 4 possible strategies are optimal, integrating over the values of *f* and *K*. The color scheme uses the CMYK color code: a purely cyan (respectively magenta, yellow) pixel indicates that the only optimal strategy for the considered values (*r, δ*) is ↓ δ (respectively ↑ *f*, ↑*K*). A color of a darker shade indicates that ↑ *r* is also optimal in a small proportion at that point, as shown on the additional color scales for each edge.

Together, our study demonstrates that bacteria can improve their association with a host by shifting their life history from a motile to a sessile, persisting lifestyle. This lifestyle shift results from correlated changes in a suite of life-history traits (Fig. 1f), which together represent a transition in life-history strategy. One way to interpret this transition is as a shift along the r–K life-history continuum, from an r-like strategy characterized by high reproductive rates to a K-like strategy characterized by persistence under high density conditions^28,29^. To demonstrate whether such a transition would generally lead to increased host association, we used an extension of a previously published mathematical model of microbial evolution towards host association^30^. Exploration of a broad parameter space with this model confirmed that increased within-host persistence is often the optimal strategy for microbial adaptation to hosts (Extended Data Fig. 10 and Supplementary Discussion), suggesting that the results from our study may be generally applicable.

In our experiments, the lifestyle shift from primarily free-living to host-associated is mediated by the Wsp system and subsequently, activity of the bacterial second messenger c-di-GMP. C-di-GMP is well known to regulate key physiological functions in bacteria, including the regulation of virulence in bacterial pathogens^22,31^. Our work demonstrates that this regulatory system promotes the adaptation of pseudomonads to diverse host systems, from plants to humans, not only in pathogens but extending to beneficial host–bacterial relationships. Given the importance of beneficial microorganisms in the functioning of their hosts, understanding the mechanisms that mediate non-pathogenic associations is crucial. Our study suggests that c-di-GMP plays an essential role in many such associations.

## Methods

### Host and bacterial strains

We performed evolution experiments with *P. lurida* strain MYb11 (*Pl*_MYb11) and its natural host *C. elegans* strain MY316 (*Ce*_MY316) (ref. ^5^). In preparation for all experiments, we thawed frozen worm stocks (−80 °C) and raised worms on nematode growth medium agar (NGM^32^) seeded with *E. coli* OP50. In additional persistence colonization experiments, we used the standard laboratory strain *C. elegans* N2 as a non-native host for the evolved bacteria. A standard bleaching protocol was used to collect sterile and synchronized L1 larvae, which were then raised to L4 stage on *E. coli* OP50 (20 °C), unless stated otherwise.

*P. lurida* strains MYb11 and MYb193, and *P. alkylphenolia* MYb187 were isolated from *Ce_*MY316 (ref. ^5^), and *P. fluorescens* SBW25 from sugar beet leaves^9^. Bacteria were cultured on tryptic soy agar (20 °C, 48 h) and tryptic soy broth (28 °C, 150 r.p.m., overnight) unless stated otherwise.

### Evolution experiment

Bacterial populations originating from a clone of *Pl*_MYb11 were serially passaged on NGM in the presence of *Ce*_MY316 (host treatment, 6 replicates) or without worms (negative control, 6 replicates). For each replicate, a lawn of *Pl*_MYb11 was seeded onto NGM and cultured for 3.5 d. For each cycle of the host treatment, 10 *C. elegans* L4 larvae were added per plate and incubated until the worms reached the F_1_ generation (3.5 d). In the negative controls, bacteria were maintained on NGM without worms. At the end of every cycle, bacteria were collected from either worms or plates in the host-associated and control treatments, respectively, 10% of the population (bottleneck) was transferred to the next cycle and a sample frozen (−80 °C). A similar number of colony-forming units (c.f.u.) was used to bottleneck the negative control. A total of 10 cycles were performed.

Frozen bacteria from cycle 10 were recovered and before further experiments were conducted, these were subjected to one more cycle of the evolution experiment to minimize any potential selective effects of freezing/thawing. To focus on evolved differences between populations of the host treatment and the negative control, rather than physiological responses to recent host exposure, bacteria were grown on NGM for 2 d as a common garden treatment and then used in subsequent assays.

### Bacterial colonization of individual worms

Bacterial fitness during host association was quantified as c.f.u.s per worm. In preparation, bacterial lawns (125 µl, optical density (OD)_600_ = 2) were seeded on NGM and 5 synchronized L4 *Ce*_MY316 added. After 3.5 d at 20 °C, worms were collected with M9 buffer containing 0.025% Triton-100 and 25 mM of the paralyzing antihelminthic tetramisole. The worms were washed in buffer using a custom-made filter tip washing system^33^ and collected in M9 with Triton-100. Worm-free supernatant was collected as a background sample. Following homogenization by bead beating, serial dilution and plating were used to quantify c.f.u.s. C.f.u.s per worm was calculated as the difference in c.f.u. between worm and supernatant samples, divided by the number of worms per population. For diversified populations, colony morphologies were scored as smooth, fuzzy or wrinkly.

### Worm population growth

Worm population growth resulting from 5 L4 larvae over 3.5 d was quantified as a proxy for host fitness. Bacteria and worms were prepared as for colonization assays and washed worms frozen in 48-well plates. Photographs of worms were automatically scored in ImageJ2 (ref. ^34^): worms were detected as particles, approximated by ellipses, and those fitting *C. elegans*-like dimensions (major axis 0.18–1.3 mm, minor axis ≤0.1 mm (ref. ^35^)) were counted. Detection quality was validated by correlating automatic worm counts with counts of two independent experimenters (*r*_(58)_ = 0.736, *P* = 2.106 × 10^−11^).

### Early colonization, persistence and release in worms

To quantify early colonization, persistence and release from L4 stage worms, bacterial lawns were prepared from ancestral *Pl*_MYb11 and evolved populations (post common garden) or clonal morphotypes (overnight cultures). In early colonization assays, we quantified bacteria that entered L4 *Ce*_MY316 that were previously raised on non-colonizing *E. coli* O50. Colonization levels were then assayed as above resulting in c.f.u.s per worm as a measure of early colonization.

For persistence and release assays, worms were raised on the respective assay bacteria (from L1 until L4 stage), mimicking the development of worms in the F_1_ generation of the evolution experiment. Worms were then collected, washed using the filter tip washing system and samples divided into supernatant (supernatant 1) and worm sample (100 µl each). Worms were then suspended in 200 µl M9 and incubated for 1 h, after which 100 µl supernatant containing released bacteria (supernatant 2) was collected. The c.f.u.s released per worm were determined by the difference in c.f.u.s between supernatant 2 and supernatant 1. Along with this, we quantified c.f.u.s maintained in worms of this sample as a measure of persistence.

### Bacterial growth, colony expansion and swarming

To measure bacterial growth, bacterial populations (common garden treatment or overnight cultures) were adjusted to OD_600_ = 0.1 and 50 µl spotted on NGM. After incubation (24 h or 3 d at 20 °C), lawns were scraped off, homogenized and c.f.u.s determined by serial dilution.

Colony expansion and swarming were assayed on NGM containing 0.5% or 3.4% agar, respectively. In either case, 0.5 µl of cell suspension (OD_600_ = 1) was spotted on surface-dried agar plates. Colony diameter was measured after 24 h, 3 and 7 d.

### Biofilm formation

In vitro biofilm formation was assayed in microtitre plates as described previously^36^. Notably, assays were performed in a randomized layout in Nunclon Delta surface-treated plates. Staining was performed after 48 h of incubation (20 °C, orbital shaking at 180 r.p.m.). Absorption of dyed biofilm solutions was measured at 550 nm using Gen5 microplate reader and Imager software (Biotek, v.3.08.01). To illustrate biofilm formation in liquid, glass test tubes were filled with 2 ml tryptic soy broth, inoculated with single colonies of ancestral *Pl*_MYb11 or evolved host-specialist mutants (*wspE*, *wspF*, *rph*) and incubated at 20 °C for 48 h until photographing.

### Isolation of morphotypes

Representative colonies with visually distinct morphologies were isolated from evolved cycle 10 populations. The evolved populations were thawed, serially diluted and plated (48 h, 20 °C). Unique morphotypes from all evolved populations were re-streaked and archived as frozen stocks (Supplementary Table 3). All morphotypes were thawed and re-streaked once, and showed stable colony morphology during 2 d of incubation.

### Growth of macrocolonies

Macrocolonies of *Pl*_MYb11 morphotypes and mutants were prepared as described previously^37^. Briefly, 5 µl of overnight culture were spotted on tryptic soy agar plates supplemented with 40 μg ml^−1^ Congo Red and incubated at 20 °C. After 24 h or 48 h, photographs were taken using a Leica fluorescence dissecting scope (LEICA M205 FA).

### Fluorescent labelling of wrinkly morphotype MT12 and in vivo microscopy

The wrinkly morphotype MT12 was labelled with red fluorescent dTomato (dT) using Tn7 transposon-based chromosomal insertion as previously described^38,39^. Insertion of the label did not affect the wrinkly morphology of the colonies.

Fluorescently labelled MT12 was used to localize colonization in *Ce*_MY316 using confocal laser scanning microscopy (ZEISS LSM 880). For this, synchronized L1 stage larvae were exposed to labelled bacteria for 72 h (20 °C), then collected using gravity washing and mounted for microscopy as previously described^39^. Overviews of complete worms were created using a ×25 LD LCI Plan-Apochromat multi-immersion objective (numerical aperture (NA) = 0.8) and details imaged using a ×40 C-Apochromat water immersion objective (NA = 1.2), in both cases using Immersol W (2010) with a refractive index of 1.334. Bacterial fluorescence and worm autofluorescence were sequentially excited (561 nm and 488 nm) and detected with an Airyscan detector (R-S sensitivity mode; longpass filter ≥570 nm; bandpass filter 495–550 nm). Data were processed with the automatic Airyscan processing function of ZEISS Efficient Navigation 2. For a list of the genetically modified bacteria used in this study, see Supplementary Table 21. After looking at the colonization of >10 worms in at least 3 biological replicate populations of consecutive weeks of experiments, a representative worm was imaged for Fig. 1e and Extended Data Fig. 2.

### Genome sequencing and analysis

Total DNA was isolated using a cetyl-trimethylammonium-bromid-based protocol^40^. For Illumina MiSeq (paired-end, 300 bp) sequencing, libraries were prepared using the Nextera DNA Flex kit. Read quality was inspected using FastQC (v.0.11.8) (ref. ^41^) and reads trimmed using Trimmomatic (v.0.3.9) (ref. ^42^). Paired reads were aligned to the *Pl*_MYb11 reference genome (RefSeq: GCF_002966835.1; Bowtie2 v.2.3.3 (ref. ^43^)) and duplicate regions removed using Picardtools (v.2.22.2) (ref. ^44^). Variants were called using BCFtools (v.1.10.2) (ref. ^45^) and VarScan (v.2.3.9) (ref. ^46^), and then annotated (snpEff^47,48^). We filtered for non-synonymous variants not present in the ancestral control in R^49,50^. Gene ontology was inferred using Pseudomonas.com^51^. To infer genes coding for enzymes with putative DGC or PDE activity, we searched for proteins with GGDEF and EAL domains using the InterProScan of the conserved domains database (CDD) via Pseudomonas.com^51^.

### Amino acid sequence alignments

To prepare amino acid sequence alignments of ancestral and mutated WspE, WspF and RPH, nucleotide sequences were translated using EMBOSS Transeq^52^ (frame 1; bacterial codon table; forward for *wspE* and *wspF*, reverse for *rph*) and resulting amino acid sequences aligned using Clustal Omega^52^ (v.1.2.4; ClustalW with character counts and standard settings). For annotation and visualization of protein domains, domain predictions of the respective sequences were collected from Pfam/InterPro (sourced from Pseudomonas.com^51^) and visually highlighted in protein visualizations prepared with DOG (v.2.0)^53^.

### Quantification of relative c-di-GMP abundances using a biosensor

To quantify intracellular concentrations of c-di-GMP in ancestral *Pl*_MYb11 and evolved wrinkly isolates (MT12: *wspF*_EVO_, MT14: *wspE*_EVO_ and MT22: *rph*_EVO_), we used an established plasmid-based biosensor^54^. Bacterial strains carrying the plasmid were grown on gentamicin-selective plates (70 h, 20 °C). For microscopy, single colonies were resuspended in 1X PBS, spotted on 2% agarose patches on microscopy slides and sealed.

Bacterial fluorescence was visualized using confocal laser scanning microscopy (ZEISS LSM 700 with ×40 Plan-Apochromat oil immersion objective (NA = 1.4) and Immersol 518F with a refractive index of 1.518). Fluorescence of the sensor and normalizer were sequentially excited (555 nm and 488 nm) and detected with a photomultiplier tube detector and a variable secondary dichroic transmitting light with wavelengths ≤630 nm and ≤550 nm, respectively. The excitation and detection settings were kept identical across all measurements.

Fluorescence intensity per cell was measured in Image J^34^: all cells and five background areas were identified as regions of interest, and area, integrated density and mean grey values were measured. Data from the untransformed images were used to calculate the corrected total cell fluorescence^55^.

In addition to single-cell measurements, we quantified c-di-GMP at the population level. For this, colonies of evolved wrinkly (MT12, MT21, MT25, MT26), smooth (MT13, MT33) and fuzzy (MT11) isolates were grown as described above, resuspended in 1X PBS and adjusted to OD_600_ = 0.1. Cell suspensions (200 µl) were then transferred to black, flat-bottomed 96-well plates with transparent bottoms (Greiner Bio-One CELLSTAR 96-well, cell culture-treated) in triplicate. After shaking (10 s, orbital shaking at 1 mm amplitude), fluorescence was sequentially excited (454 nm and 460 nm, bandwidth 9 nm, 10 flashes) and emission detected (585 nm and 510 nm, bandwidth 20 nm; optimal gain, 20 µs integration) in a plate reader (Tecan, Infinite M200Pro), with 1X PBS serving as the background control.

To infer c-di-GMP concentration, we calculated the relative fluorescence intensity, or the ratio between TurboRFP and AmCyan fluorescence intensities, as previously described^54^, and compared average relative fluorescence intensities between ancestral *Pl_*MYb11 and evolved wrinkly, smooth and fuzzy morphotypes. For the images used in Fig. 2, linear LUT was used at full range. Brightness and contrast were applied equally to all images.

### Quantification of c-di-GMP using parallel reaction monitoring LC–MS/MS

To quantify intracellular c-di-GMP using LC–MS in parallel reaction monitoring mode, ancestral and evolved *Pl*_MYb11 (MT12, MT14 and MT22) were grown in LB medium to an OD_600_ of 1.8 and pelleted by centrifugation. After washing with salt-free LB medium, pelleted cells were snap frozen and stored (−80 °C). Cells were mixed with 10 pmol of internal standard (cyclic-di-GMP-^13^C_20_,^15^N_10_, Toronto Research Chemicals) in 60 μl of water. Extraction of c-di-GMP was performed as previously described^56^ with the following modifications: extraction solution (240 μl of 1:1 acetonitrile (ACN)/methanol (MeOH)) was added and samples were vigorously vortexed. Following incubation on ice (15 min) and centrifugation (20,800 × *g*, 4 °C, 2 min), extract supernatant was collected and solvent extraction repeated twice (200 μl of 2:2:1 ACN/MeOH/water). Pooled extracts were dried, resuspended in 50 μl of water and centrifuged to remove insoluble compounds. Concentrations of solubilized protein precipitates were determined using the Pierce BCA protein assay kit (Thermo Fisher). For LC–MS/MS, 1 μl extract was injected onto an EASY-nLC 1000 UHPLC (Thermo Fisher) and separated on a 15-cm ReproSil-Pur C_18_-AQ nano LC column (0.1 mm i.d., 1.9 μm, 120 Å, Altmann Analytik) at 400 nl min^−1^. Eluent A was 10 mM NH_4_OAc with 0.1% HAc, eluent B was 100% MeOH. Chromatographic conditions were 5% eluent B (5 min), followed by a linear gradient from 5% to 20% B (15 min) and an increase to 70% B (1 min), followed by 70% B (5 min) and 5% B (5 min); higher-energy collisional dissociation of the *m*/*z* 691.1021 and *m*/*z* 721.0714 precursors was performed on a Q Exactive HF Orbitrap MS (Thermo Fisher). Peak areas for the qualifying^57^ product ions *m*/*z* 248.0778 (light) and *m*/*z* 263.0965 (heavy) determined in Skyline (v.21.1.0.146.3, MacCoss Lab software)^58^ were used to calculate total c-di-GMP amounts, which were normalized to total protein amount as obtained by the BCA assay.

### Mutant generation

A two-step allelic replacement method based on previously described protocols^21,59^ was used to introduce the evolved mutant alleles into an ancestral background and also to revert mutations by introducing ancestral alleles in the mutant background. We applied the following modifications: ~700 bp long PCR amplicons surrounding each mutation were cloned into pUISacB allowing for sucrose selection. The constructs were transformed into competent *E. coli* cells and transferred to *Pseudomonas* isolates via conjugative mating with an *E. coli* helper strain containing pRK2013 (ref. ^60^). Primers (see Supplementary Table 22) were designed using NCBI’s BLAST tool^61^ and NCBI Primer-BLAST^62^, NEBuilder v.2.3.0 (New England Biolabs) and Oligo Analyse Tool (Eurofins Genomics). BLASTn and alignments with Clustal Omega^63^ were performed using default settings.

### Heterologous expression of phosphodiesterase and diguanylate cyclase

To manipulate intracellular c-di-GMP levels and study the consequences for host association and colony morphology, we expressed a heterologous PDE and a heterologous DGC in our evolved host-specialist mutants (*wspE, wspF* and *rph*). The PDE PA2133 from *P. aeruginosa* was expressed from plasmid pJN2133 (ref. ^23^). A constitutively active GCN4-WspR fusion construct^27^ was synthesized (Eurofins) and then cloned into pJStrep to generate a C-terminal StrepII-tagged GCN4-WspR construct. Empty pJStrep (a modified pJN105 (ref. ^64^) vector containing the StrepII tag coding sequence) and empty pJN105 plasmids were used as controls. All plasmids were introduced into *Pl*_MYb11 and evolved mutants using a previously described electroporation protocol^65^.

### In vivo competition assays

Competition experiments were performed as described for the short-term persistence assays. Co-inoculated bacteria were OD-adjusted and mixed in equal volumes before seeding as lawns on NGM agar. A *Pl*_MYb11 labelled with dTomato^39^ was used, which is equivalent to the ancestral *Pl*_MYb11, as no differences were observed in short-term persistence (analysis of variance (ANOVA), *F* value = 0.99, d.f. = 1, *P* = 0.35). C.f.u.s per worm were determined by subtracting c.f.u.s in supernatants from those in worm samples. A competitive index was calculated as the ratio of c.f.u.s per worm of evolved or constructed mutants to c.f.u.s per worm of the ancestor.

### Correlation of *wsp* and *rph* gene presence with isolate source across pseudomonads

Whole-genome sequences from NCBI were mined for c-di-GMP modulating genes (focus: *wsp* operon, *rph*) with bacterial lifestyle in members of the genus *Pseudomonas*. First, candidate genomes were obtained (NCBI Nucleotide’s command line search tool; size: 5–8 million bp). This retrieved 2,279 sequences, for which sample information from NCBI’s Biosample database was collected. When available, host, host disease status, isolation source and sample type were used to manually classify genomes as originating from free-living or host-associated isolates with or without/unknown disease (Supplementary Table 18). Next, we downloaded all available *Pseudomonas* reference sequences for *rph* and *wsp* genes from pseudomonas.com^51^. These were used to identify candidate sequences of *rph*, *wspA*, *wspB*, *wspC*, *wspD*, *wspE*, *wspF* and *wspR*. These target gene candidates were found in the selected genomes using BLAST (R package ‘rBLAST’) and filtered on the basis of sequence lengths and percent identities of the BLAST hits (Extended Data Fig. 9). Percent identity and sequence length were selected to maximize the chance that genes were correctly identified (red rectangles in Extended Data Fig. 9). If at least one candidate gene was identified during BLAST searches with the reference genes as query, this gene was considered present in the respective genome. We then used *χ*^2^ goodness-of-fit tests to infer whether isolates with and without the target genes differed in the relative proportions of host-associated lifestyles (Supplementary Table 19).

### Detection of signatures of selection

To assess whether our focal host-specialist genes (*wspE, wspF, rph*) were experiencing positive or purifying selection in the genus *Pseudomonas*, we performed MUSCLE codon-based multiple sequence alignments of nucleotide sequences (see dataset described above) using MEGA11 (ref. ^66^; default settings). Subsequently, we performed codon-based *z*-tests (default settings) to test for significant deviations from neutral selection. In addition, we analysed signatures of selection in Blast hits for a set of three *Pseudomonas* core genes (*gyrB:* PA0004, *rpoD:* PA0576 and a 16S rRNA methyltransferase: PA0419; see also ref. ^67^) in the set of genomes studied for *wsp* and *rph* presence/absence, also using multiple sequence alignments and codon-based tests of neutrality.

### Statistical analyses

Before data collection, no statistical methods were used to pre-determine samples sizes, but our sample sizes are similar to those reported in previous publications. In all experiments, treatments and samples were blinded and randomized. Before data analysis, assumptions of parametric models (normality, homogeneity of variances) were checked by visual inspection (box-/qqplots) and with Shapiro–Wilk and Levene tests. When these were not met, non-parametric tests were applied. Boxplots show median (centre line), upper/lower quartiles (box limits) and 1.5× interquartile ranges (whiskers).

To check whether evolved populations differed from the ancestor in c.f.u.s per worm, we compared the shift in the evolved phenotype (ratio of c.f.u.s per worm of evolved populations to those of ancestral *Pl*_MYb11) to the ancestral phenotype using one-sample *t*-tests (alpha = 0.05, mu = 1) with false discovery rate (FDR^68^) correction for multiple testing. We applied this approach to analyse: bacterial colonization of individual worms, worm population growth, early colonization, persistence and release, colony expansion and swarming. To infer overall phenotypic shifts according to evolutionary treatment, a principal component analysis (PCA) including the assayed phenotypes was performed. We performed permutational analysis of variances (PERMANOVA, 1,000 permutations) followed by pairwise comparisons of groups (FDR-corrected) to test for differences in phenotype sets of ancestral and evolved groups, and plotted confidence ellipses (one standard deviation). Packages used included ggbiplot^69^, missMDA^70^, vegan^71^ and pairwise.adonis^72^.

Differences in proportions of the different colony morphologies (wrinkly, smooth and fuzzy) within worms were identified using generalized linear models (GLM; quasinormal distribution) with Tukey post hoc tests (using lme4 (ref. ^73^), lmtest^74^ and multcomp^75^).

Changes in morphotype proportions over time were tested using beta-regressions (using gamlss^76^).

Differences between morphotype phenotypes were detected using ANOVA or GLMs, followed by Tukey or Dunnett post hoc tests. To infer functional specializations across phenotypes, we used PCA and PERMANOVA.

Differences in biofilm formation and motility between evolved wrinkly host specialists (*wspE*, *wspF* and *rph* mutants) and ancestral *Pl*_MYb11 were analysed using nested ANOVAs followed by Tukey post hoc tests. When no batch effects (evolved population of origin) were detected, mutants were compared across populations. In the case of swarming diameters, however, the *rph* mutant was compared to the co-analysed ancestral *Pl*_MYb11 using a *t*-test.

Differences in c-di-GMP concentrations between evolved isolates were inferred using Welch’s ANOVA, nested ANOVA or ANOVA with Games–Howell or Dunnett post hoc comparisons.

We tested for differences in c.f.u.s per worm between morphotypes or mutants using GLMs and linear mixed models (LMMs), and Dunnett or Tukey post hoc comparisons.

All analyses and plotting were performed in R^49,50,77,78^.

### Mathematical model

We built a model to assess the selection gradient experienced by bacteria during the evolution experiment (Extended Data Fig. 10). We focused on the phase when bacteria are in contact with worms and considered a homogeneous population. The dynamics of the number of bacteria living in (any) host association *n(t)* can be described by the equation

1 $$\frac{{dn}(t)}{{dt}}=f\,W\left(t\right)+r\,n\left(t\right)\left(1-\frac{n\left(t\right)}{K\,W\left(t\right)}\right)-\delta n(t),$$

where *W(t)* denotes the biomass of worms on the plate at time *t*. We consider that growing to saturation, bacteria on the plate are always in excess so that only the number of worms and the rate *f* at which they feed on bacteria limit the immigration of free-living bacteria to the host. We assume logistic growth of the bacterial population within the worms, with maximal rate *r* and a carrying capacity proportional to the biomass of worms *W*(*t*) and the per unit of worm biomass carrying capacity *K*. Finally, a fraction of the host-associated bacterial population is removed from the host at a rate *δ*, which encompasses bacterial death and expulsion to the environment. As in the evolution experiment, we assume that only host-associated bacteria are selected and continue to the next cycle, ignoring on-plate dynamics. We assume linear growth for the worm biomass, *W*(*t*) = *g t* + *W*_*0*_, encompassing both reproduction and development. We neglect the potential evolution of beneficial effects on worm growth and fix the parameters *W*_0_ = 10 and *g* = 711 d^−1^ to experimentally observed values.

We studied how the final number of host-associated bacteria, *n*_f_, is affected by changes in the parameters that describe the bacterial life cycle (*r, δ, f, K*). We defined a range of biologically plausible values for each of these parameters (that is, the trait space) that are informed by experimental data where possible:

- 10^−1^ d^−1^ < *r* < 10^1.25^ d^−1^, that is, between a small fraction and around twice the maximum on-plate growth rate (~7 d^−1^).
- 10^−0.5^ d^−1^ < *δ* < 10^4^ d^−1^, as the typical time for a worm to lose 50% of its microbiome (in the absence of feeding and replication) should range between seconds and days.
- 10^4^ < *K* < 10^6.25^, given the orders of magnitude from the maximal number of bacteria per worm measured experimentally (~10^5^).
- 10^3^ d^−1^ < *f* < 10^7.5^ d^−1^, as the typical time for an empty worm to be colonized at 10% of its carrying capacity (*K* = 10^5^) should vary between seconds and days, neglecting bacterial release and within-host replication.

For each point of the trait space, we numerically solved equation (1) to compute the expected final number of bacteria at *t*_f_ = 3.5 d, *n*_f_ = *n(t*_f_). Finally, we assessed the elasticity of *n*_f_ along each direction of the trait space, which measures the expected relative change in *n*_f_ with respect to a small relative change in one of the traits. We interpreted the vector of the elasticities as the selection gradient on the phenotypic traits^79^ and used the dominant element of this vector to define an ‘optimal evolution strategy’^30^ for each point of the trait space.

### Reporting summary

Further information on research design is available in the Nature Portfolio Reporting Summary linked to this article.

### Supplementary information

Reporting Summary

Supplementary Tables Supplementary Tables 1–22.

## Data Availability

Raw sequencing data are available at NCBI under Bioproject PRJNA862108. All other data are accessible at https://github.com/nobeng/c-di-GMP_host-association.

## Code availability

Custom code and associated data are available at https://github.com/nobeng/c-di-GMP_host-association.
