# Supplementary material for: Bacterial c-di-GMP has a key role in establishing host–microbe symbiosis
Source: Nat Microbiol. 2023 Aug 31;8(10):1809–19. doi: 10.1038/s41564-023-01468-x (PMC10522488; doi:10.1038/s41564-023-01468-x)
Supplement: Supplementary file 1 — Reporting Summary [file 41564_2023_1468_MOESM1_ESM.pdf]

nature portfolio

Corresponding author(s): Hinrich Schulenburg

Last updated by author(s): 01/08/2023

Reporting Summary

Nature Portfolio wishes to improve the reproducibility of the work that we publish. This form provides structure for consistency and transparency in reporting. For further information on Nature Portfolio policies, see our Editorial Policies and the Editorial Policy Checklist.

Statistics

For all statistical analyses, confirm that the following items are present in the figure legend, table legend, main text, or Methods section.

n/a	Confirmed	
☐	☒	The exact sample size (*n*) for each experimental group/condition, given as a discrete number and unit of measurement
☐	☒	A statement on whether measurements were taken from distinct samples or whether the same sample was measured repeatedly
☐	☒	The statistical test(s) used AND whether they are one- or two-sided<br>*Only common tests should be described solely by name; describe more complex techniques in the Methods section.*
☐	☒	A description of all covariates tested
☐	☒	A description of any assumptions or corrections, such as tests of normality and adjustment for multiple comparisons
☐	☒	A full description of the statistical parameters including central tendency (e.g. means) or other basic estimates (e.g. regression coefficient) AND variation (e.g. standard deviation) or associated estimates of uncertainty (e.g. confidence intervals)
☐	☒	For null hypothesis testing, the test statistic (e.g. F, t, r) with confidence intervals, effect sizes, degrees of freedom and P value noted<br>*Give P values as exact values whenever suitable.*
☒	☐	For Bayesian analysis, information on the choice of priors and Markov chain Monte Carlo settings
☐	☒	For hierarchical and complex designs, identification of the appropriate level for tests and full reporting of outcomes
☐	☒	Estimates of effect sizes (e.g. Cohen's *d*, Pearson's *r*), indicating how they were calculated

Our web collection on statistics for biologists contains articles on many of the points above.

Software and code

Policy information about availability of computer code

Data collection	Absorption data: Gen5 Microplate Reader and Imager Software (v3.08.01; Biotek) and Tecan Microplate Reader (Infinite M200Pro)<br>Confocal microscopy: ZEISS Efficient Navigation 2 (Carl Zeiss Microscopy GmbH)<br>LC-MS/MS: Skyline v21.1.0.146.3 (MacCoss Lab Software)<br>Genome survey: NCBI Nucleotide, NCBI's Biosample database; R packages: rentrez, rBLAST
Data analysis	Genomic data was analyzed using the following softwares and packages: Python v3.7.4, FastQC v0.11.8, Trimmomatic v0.3.9, Bowtie2 v2.3.3, Picardtools v2.22.2, samtools v1.5, BCFtools v1.10.2, zlib v1.2.11, bzip2 v2-1.0.6, xz Utils v5.2.4, VarScan v2.3.9, curl v7.68.0, snpEff v4.3, R (v4.1.0, v4.1.3 and v4.3.1).<br>Image data was analyzed using ImageJ2 (v1.53k).<br>Protein domains were visualized using DOG 2.0.<br>Statistical analyses were performed in R Studio (R v4.1.0, v4.1.3 and v4.3.1) using ggbiplot, missMDA, vegan, lme4, lmtest, multcomp, gamlss.<br>Mathematical modeling was performed in Mathematica (v12.1.1.6958981).

For manuscripts utilizing custom algorithms or software that are central to the research but not yet described in published literature, software must be made available to editors and reviewers. We strongly encourage code deposition in a community repository (e.g. GitHub). See the Nature Portfolio guidelines for submitting code & software for further information.

Data

Policy information about availability of data

All manuscripts must include a data availability statement. This statement should provide the following information, where applicable:

- Accession codes, unique identifiers, or web links for publicly available datasets
- A description of any restrictions on data availability
- For clinical datasets or third party data, please ensure that the statement adheres to our policy

Custom code and corresponding data sheets are available at https://github.com/nobeng/c-di-GMP_host-association. Raw sequencing data is available in the NCBI Bioproject PRJNA862108.

The following databases were used in this study: Pseudomonas.com, Conserved Domains Database (CDD; https://www.ncbi.nlm.nih.gov/Structure/cdd/cdd.shtml), NCBI BioSample (https://www.ncbi.nlm.nih.gov/biosample), Pfam and InterPro (https://www.ebi.ac.uk/interpro/), and RefSeq: NCBI Reference Sequence Database (https://www.ncbi.nlm.nih.gov/refseq/; to obtain the Pseudomonas lurida MYb11 reference genome: GCF_002966835.1)

Human research participants

Policy information about studies involving human research participants and Sex and Gender in Research.

Reporting on sex and gender	NA
Population characteristics	NA
Recruitment	NA
Ethics oversight	NA

Note that full information on the approval of the study protocol must also be provided in the manuscript.

Field-specific reporting

Please select the one below that is the best fit for your research. If you are not sure, read the appropriate sections before making your selection.

☐ Life sciences ☐ Behavioural & social sciences ☒ Ecological, evolutionary & environmental sciences

For a reference copy of the document with all sections, see nature.com/documents/nr-reporting-summary-flat.pdf

Ecological, evolutionary & environmental sciences study design

All studies must disclose on these points even when the disclosure is negative.

Study description	Experimental evolution of Pseudomonas lurida MYb11 to host-association with C. elegans, whole genome sequence analysis, bacterial functional genetics and molecular analysis, in vitro and in vivo competition experiments with different Pseudomonads, and comparative bioinformatics.
Research sample	Pseudomonas lurida strain MYb11 and its evolved derivatives, Caenorhabditis elegans strain MY316, Pseudomonas lurida strain MYb193, Pseudomonas alkylphenolia strain MYb187, Pseudomonas fluorescens strain SBW25, Escherichia coli OP50
Sampling strategy	An evolution experiments was performed including 2 treatments (host-associated, no host control) and 6 replicate populations per treatment. The evolution experiment was performed by serially passaging P. lurida MYb11 in a host-associated life cycle (with worms and on agar) or a no-host (agar only) control life cycle. Passaging was performed for 10 cycles.
Data collection	Evolving populations were sampled at each cycle, and evolved materials characterized in detail after recovery from frozen stocks. Experimentors involved in the study are detailed in author contributions.
Timing and spatial scale	Evolving populations were sampled at each cycle to track evolutionary dynamics and changes of evolved populations from cycle 10 compared to ancestral P. lurida MYb11 were characterized in detail.
Data exclusions	All data points were included in our analysis. For worm colonization experiments (incl. early colonization, persistence, release) negative CFU/worm values were excluded. Two bacterial isolates from worms that were identified as E. coli OP50, which served as a food bacterium for C. elegans, were excluded from detailed genomic analyses.
Reproducibility	An evolution experiment with 6 independent replicates per treatment was performed. Characterization of evolved isolates and follow-up experiments were done at least in triplicate.
Randomization	Samples were always randomized during experiments. For colonization assays, replicate evolved populations were sampled

Randomization	sequentially (always including an ancestral control and both treatments). Accordingly, replicate was included as a covariate in the respective statistical models.
Blinding	Treatment groups in all experiments were masked to minimize observer bias.

Did the study involve field work? ☐ Yes ☒ No

Reporting for specific materials, systems and methods

We require information from authors about some types of materials, experimental systems and methods used in many studies. Here, indicate whether each material, system or method listed is relevant to your study. If you are not sure if a list item applies to your research, read the appropriate section before selecting a response.

Materials & experimental systems

n/a	Involved in the study
☒	☐ Antibodies
☒	☐ Eukaryotic cell lines
☒	☐ Palaeontology and archaeology
☐	☒ Animals and other organisms
☒	☐ Clinical data
☒	☐ Dual use research of concern

Methods

n/a	Involved in the study
☒	☐ ChIP-seq
☒	☐ Flow cytometry
☒	☐ MRI-based neuroimaging

Animals and other research organisms

Policy information about studies involving animals; ARRIVE guidelines recommended for reporting animal research, and Sex and Gender in Research

Laboratory animals	Caenorhabditis elegans strain MY316 (L4 larvae -> young adults) were used.
Wild animals	No wild animals were used in this study.
Reporting on sex	All experiments were performed with hermaphrodites.
Field-collected samples	No field-collected samples were used in this study.
Ethics oversight	No ethical oversight organization was necessary for use of C. elegans.

Note that full information on the approval of the study protocol must also be provided in the manuscript.
